# Total tumour volume as a prognostic factor in patients with resectable colorectal cancer liver metastases

**DOI:** 10.1002/bjs5.50280

**Published:** 2020-04-11

**Authors:** K. Tai, S. Komatsu, K. Sofue, M. Kido, M. Tanaka, K. Kuramitsu, M. Awazu, H. Gon, D. Tsugawa, H. Yanagimoto, H. Toyama, S. Murakami, T. Murakami, T. Fukumoto

**Affiliations:** ^1^ Department of Surgery Division of Hepato‐Biliary‐Pancreatic Surgery Kobe Hyogo Japan; ^2^ Department of Radiology Kobe University Graduate School of Medicine Kobe Hyogo Japan; ^3^ Clinical and Translational Research Centre, Kobe University Hospital Kobe Hyogo Japan

## Abstract

**Background:**

Although total tumour volume (TTV) may have prognostic value for hepatic resection in certain solid cancers, its importance in colorectal liver metastases (CRLM) remains unexplored. This study investigated its prognostic value in patients with resectable CRLM.

**Method:**

This was a retrospective review of patients who underwent hepatic resection for CRLM between 2008 and 2017 in a single institution. TTV was measured from CT images using three‐dimensional construction software; cut‐off values were determined using receiver operating characteristic (ROC) curve analyses. Potential prognostic factors, overall survival (OS) and recurrence‐free survival (RFS) were determined using multivariable and Kaplan–Meier analyses.

**Results:**

Some 94 patients were included. TTV cut‐off values for OS and RFS were 100 and 10 ml respectively. Right colonic primary tumours, primary lymph node metastasis and bilobar liver metastasis were included in the multivariable analysis of OS; a TTV of 100 ml or above was independently associated with poorer OS (hazard ratio (HR) 6·34, 95 per cent c.i. 2·08 to 17·90; *P* = 0·002). Right colonic primary tumours and primary lymph node metastasis were included in the RFS analysis; a TTV of 10 ml or more independently predicted poorer RFS (HR 1·90, 1·12 to 3·57; *P* = 0·017). The 5‐year OS rate for a TTV of 100 ml or more was 41 per cent, compared with 67 per cent for a TTV below 100 ml (*P* = 0·006). Corresponding RFS rates with TTV of 10 ml or more, or less than 10 ml, were 14 and 58 per cent respectively (*P* = 0·009). A TTV of at least 100 ml conferred a higher rate of unresectable initial recurrences (12 of 15, 80 per cent) after initial hepatic resection.

**Conclusion:**

TTV was associated with RFS and OS after initial hepatic resection for CRLM; TTV of 100 ml or above was associated with a higher rate of unresectable recurrence.

## Graphical abstract

This study investigated whether the total tumour volume (TTV) of colorectal liver metastases could predict patient survival after hepatic resection. Associations were identified between TTV and recurrence‐free survival, overall survival and unresectable recurrence after primary hepatic resection. TTV may represent a good basis for determining indications for hepatic resection.

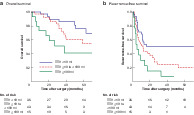

Total tumour volume predicts both overall and recurrence‐free survival

## Abstract (es)

**Antecedentes:**

Aunque el volumen total del tumor (total tumour volume, TTV) puede tener valor pronóstico tras la resección hepática (hepatic resection, HR) en algunas neoplasias sólidas, no se conoce su importancia en las metástasis hepáticas de cáncer colorrectal (colorectal liver metastases, CRLMs). Este estudio analizó el valor pronóstico del TTV en pacientes con CRLMs resecables.

**Métodos:**

Revisión retrospectiva de pacientes a los que se realizó una HR por CRLMs entre 2008 y 2017 en un solo centro. El TTV se estimó a partir de imágenes de tomografía computarizada utilizando un programa de reconstrucción 3D; se determinaron los valores de corte mediante un análisis de las características operativas del receptor. Se identificaron los posibles factores pronósticos y se calcularon la supervivencia global (*overall survival*, OS) y la supervivencia libre de recidiva (*recurence‐free survival*, RFS) mediante análisis multivariados y de Kaplan‐Meier.

**Resultados:**

Se incluyeron 94 pacientes. Los valores de corte del TTV para la OS y la RFS fueron de 100 mL y 10 mL, respectivamente. En el análisis multivariable para la OS, se incluyeron los tumores del colon derecho, las metástasis linfáticas primarias y la metástasis hepática bilobar; un TTV ≥ 100 mL se asoció de forma independiente con una peor OS (cociente de riesgos instantáneos, *hazard ratio*, HR, 6,34, i.c. del 95% 2,08‐17,9; *P* = 0,002). En el anáisis para la RFS, se incluyeron tumores primarios de colon derecho y las metástasis linfáticas primarias; un TTV ≥ 10 mL predijo de forma independiente una peor RFS (HR 1,90, i.c. del 95% 1,12‐3,57; *P* = 0,017). Las tasas de OS a los 5 años con TTVs ≥ 100 mL *versus* < 100 mL fueron del 41% *versus* 67% (*P* = 0,006); las tasas de RFS respecto a TTVs ≥ 10 mL *versus* < 10 mL fueron del 14% *versus* 58% (*P* = 0,009). Un TTV ≥ 100 mL conllevó una tasa más elevada (80%) de recidivas no resecables después de la HR inicial.

**Conclusión:**

El TTV se asoció con la RFS y la OS tras la HR por CRLMs; unos valores ≥ 100 mL conllevan una tasa más elevada de recidiva irresecable.

## Introduction

Colorectal cancer is among the most common causes of cancer‐related deaths worldwide^1^, ^2^, and the liver is the commonest site of distant metastasis^3^, ^4^, ^5^. Hepatic resection is generally regarded as the most effective treatment and the only potentially curative intervention for colorectal liver metastases (CRLM)^4^, ^6^. Current standard treatments for patients with unresectable or difficult‐to‐resect CRLM include neoadjuvant chemotherapy^7^, ^8^, ^9^. This modality may be able to shrink the tumour, thereby reducing the volume of the liver requiring resection, or even render an initially unresectable CRLM resectable^10^.

Primary tumour factors (such as tumour site, invasiveness of the lesion and lymph node metastasis status) and preoperative factors (such as carcinoembryonic antigen (CEA) level, number of metastases and tumour size) are associated with patient outcomes after hepatic resection for CRLM^11^, ^12^, ^13^, ^14^, ^15^, ^16^. Most preoperative factors may be related to the total tumour volume (TTV) in the liver. In this regard, recent advances in diagnostic imaging enable direct measurement of the TTV, and have indicated an association between TTV and prognosis for patients with many types of solid tumour, including hepatocellular carcinoma, which is the most prevalent liver cancer^17^, ^18^, ^19^. In patients with hepatocellular carcinoma who underwent liver transplantation or hepatic resection, TTV has been reported as a good predictor of recurrence‐free (RFS) and overall (OS) survival^20^, ^21^, ^22^, ^23^, ^24^. However, the association between TTV and prognosis in patients with CRLM has not been studied.

Previously, combinations of prognostic factors, such as maximum tumour diameter and number of metastases, have been used to determine the indication for hepatic resection in patients with CRLM. Several prognostic scores, such as the Tumour Burden Score (TBS) and Fong score, have been described^25^, ^26^. The indication for hepatic resection has subsequently been expanded to include any patient with CRLM in whom all tumour lesions can be removed with a negative margin and who retain an adequate remnant liver volume^27^. More recently, new strategies such as portal vein embolization, two‐stage hepatectomy, and associating liver partition and portal vein ligation for staged hepatectomy (ALPPS) have been introduced to increase the hepatic function reserve and expand the pool of candidates for hepatic resection^28^, ^29^. However, the indication for hepatic resection in patients with huge and/or multiple CRLM remains debated.

The present study aimed to evaluate the association between TTV and prognosis in patients with resectable CRLM, and also to investigate the value of TTV as an indicator for hepatic resection.

## Methods

Consecutive patients with CRLM who underwent hepatic resection between April 2008 and September 2017 at Kobe University Hospital were initially considered eligible for this retrospective cohort study. Patients who met one or more of the following criteria were excluded: did not undergo initial hepatic resection at Kobe University Hospital; extrahepatic metastasis detected before initial hepatic resection; incomplete resection (gross residual tumour); two‐stage hepatectomy performed; and hepatic resection performed after liver transplantation.

Informed consent was obtained using an opt‐out form. The requirement for individual consent was waived owing to the retrospective study design. This study complied with the standards of the Declaration of Helsinki, as well as current ethical guidelines, and was approved by the institutional ethics board of Kobe University on 19 March 2019 (number 180366).

### Outcome

The ability of the TTV to predict prognosis was evaluated based on the OS and RFS after initial hepatic resection. Patients who were lost to follow‐up were censored on the date of the last contact. Patients who were still alive on 30 December 2018 were censored on that date for the OS analysis. Those who had experienced no disease recurrence by the same date were also censored for the RFS analysis. RFS and OS were calculated from the date of the initial hepatic resection to the date of disease recurrence and to the date of death from any cause respectively.

### Indication for treatment

Eligibility criteria for hepatic resection for CRLM were as follows: technically resectable tumours involving no more than three hepatic segments; preserved liver function and indocyanine green retention rate at 15 min (ICGR15) of less than 25 per cent; residual functional volume of the liver greater than 30 per cent of the standard liver volume; no main portal vein trunk involvement or distant metastasis; and an Eastern Cooperative Oncology Group performance status score of 0–2.

All patients in whom hepatic resection was indicated underwent the procedure as a first‐line treatment, regardless of tumour number, tumour size or timing of metastasis (synchronous or metachronous). Partial hepatic resection was performed with a resection margin greater than 0 mm. Anatomical hepatic resection was performed according to the tumour size and location, as appropriate. Patients with initially unresectable CRLM received chemotherapy; those who achieved downstaging to resectable status (tumour shrinkage and satisfactory surgical criteria) underwent hepatic resection. Surgical resection was indicated for both intrahepatic and extrahepatic (lung or lymph node) recurrent lesions that were detected after hepatic resection and deemed to be resectable. Unresectable recurrent lesions were treated with chemotherapy or radiotherapy to the extent possible.

### Data collection

Patient data were collected from electronic medical records. Patient demographics, preoperative imaging findings, preoperative and postoperative treatments, response to preoperative chemotherapy, Model for End‐Stage Liver Disease (MELD) scores^30^, ICGR15, and CEA and carbohydrate antigen (CA) 19‐9 levels before hepatic resection were recorded. Response to preoperative chemotherapy was assessed according to Response Evaluation Criteria In Solid Tumours (RECIST) criteria, version 1.1^31^. CRLM were initially diagnosed using dynamic contrast‐enhanced (DCE) CT (DCE‐CT), with acquisition of precontrast, arterial, portal venous and equilibrium phase images. Ancillary MRI and/or biopsy were also done if the CT findings were equivocal.

### Calculation of total tumour volume

**Figure 1:**
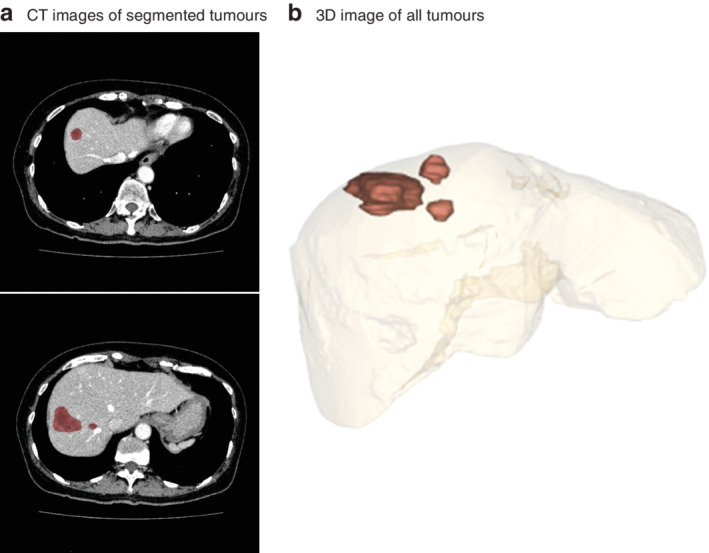
Total tumour volume assessment using Ziostation2® (Ziosoft, Tokyo, Japan) software **a** CT images of segmented tumours using the software sculpting tool. **b** Three‐dimensional (3D) image of all the tumours included in the total tumour volume measurement.

Tumour volumes were measured in all patients; Ziostation2® (Ziosoft, Tokyo, Japan) software was used to construct three‐dimensional images based on the original preoperative DCE‐CT image (*Fig*. 1). Each imaging set was reviewed, and the contrast image phase (arterial or portal venous) that provided the clearest distinction between tumour and background liver tissue was selected. The software sculpting tool was used to segment each tumour and provide a measure of the tumour volume in millilitres. The TTV was then calculated as the sum of the volumes of all tumours in an individual patient.

### Definition of total tumour volume cut‐off value

The TTV cut‐off values that would best predict OS and RFS were determined using receiver operating characteristic (ROC) curve analysis, in which 3‐year OS and RFS rates were used as objective variables. Patients whose observation period did not reach 3 years were excluded.

### Analysis of survival and recurrence after hepatic resection based on total tumour volume

To investigate the association between RFS and OS, patients were categorized into three groups according to TTV cut‐off values for RFS and OS. RFS, OS and status of the first recurrence after hepatic resection were compared among these three groups.

### Statistical analysis

All statistical analyses were two‐tailed, and the threshold for significance was *P* < 0·050. Continuous data are presented as median (i.q.r.) values, and categorical data are summarized as frequencies and percentages. Univariable and multivariable analyses of prognostic factors were performed using the Cox proportional hazards model. Baseline variables with *P* < 0·050 in univariable analysis were included in the multivariable model. To compare the prognostic power of TTV with the TBS and Fong scores, multivariable analysis was performed using either TBS or Fong score instead of TTV. Each score was analysed using the same independent variables as for TTV. Survival was estimated using the Kaplan–Meier method, and survival estimates were compared with the log rank test. The χ^2^ test was used to compare the number of postoperative recurrences. All statistical analyses were conducted using JMP® 13 software (SAS Institute, Cary, North Carolina, USA).

## Results

**Figure 2:**
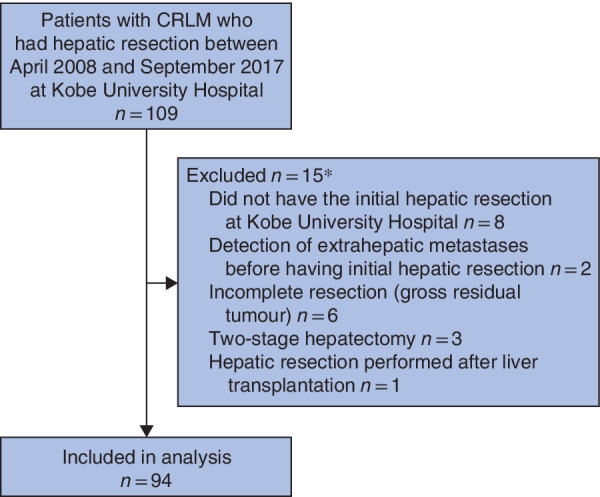
Flow diagram for the study *Five patients met two exclusion criteria. CRLM, colorectal liver metastases.

A total of 109 consecutive patients with CRLM who underwent hepatic resection were initially considered eligible. After exclusions, 94 patients were included in the study (*Fig*. 2); their baseline characteristics are shown in *Table* 1.

**Table 1:** Baseline characteristics of patients with colorectal liver metastases who underwent hepatic resection at Kobe University Hospital

	No. of patients* (*n* = 94)
**Age (years)** †	67 (61–74)
**Sex ratio (M** : **F)**	65 : 29
**CEA level ≥ 200 ng/ml**	12 (13)
**CA19‐9 level ≥ 37 units/ml**	35 (37)
**MELD score** †	0·87 (−2·7 to 3·6)
**ICGR15 > 10%**	29 of 88 (33)
**Primary tumour**	
Right colonic location	23 (24)
Lymphatic invasion	48 of 86 (56)
Vessel invasion	61 (65)
Lymph node metastasis	56 of 92 (61)
**Metastases**	
Maximum diameter (cm)†	3·4 (2·1–5·4)
Diameter ≥ 5 cm	29 (31)
No. of tumours†	1 (1–3)
≥ 5	14 (15)
Total tumour volume (ml)†	14 (4·8–55)
Bilobar tumour distribution	27 (29)
Residual tumour	17 of 93 (18)
Preoperative chemotherapy	47 (50)
RECIST response to preoperative chemotherapy	*n* = 47
Partial response	24 (51)
Stable disease	23 (49)
Postoperative therapy	59 of 90 (66)
Lobectomy	27 (29)

* With percentages in parentheses unless indicated otherwise;

† values are median (i.q.r.). CEA, carcinoembryonic antigen; CA, carbohydrate antigen; MELD, Model for End‐stage Liver Disease; ICGR15, indocyanine green retention rate at 15 min; RECIST, Response Evaluation Criteria In Solid Tumours.

The median follow‐up was 35 (range 17–60) months, and median patient age was 67 years. Half of the patients had preoperative chemotherapy, and 66 per cent (59 of 90) received postoperative chemotherapy and/or radiotherapy. Perioperative chemotherapy regimens varied; however, all administered treatments were considered the best available at that time. The median TTV of the metastatic liver lesions was 14 ml, and 40 patients had multiple liver metastases. The 1‐, 3‐ and 5‐year OS rates of patients in this study were 93, 74 and 60 per cent respectively; corresponding RFS rates were 49, 32 and 32 per cent.

### Determination of total tumour volume cut‐off values and survival based on these values

**Figure 3:**
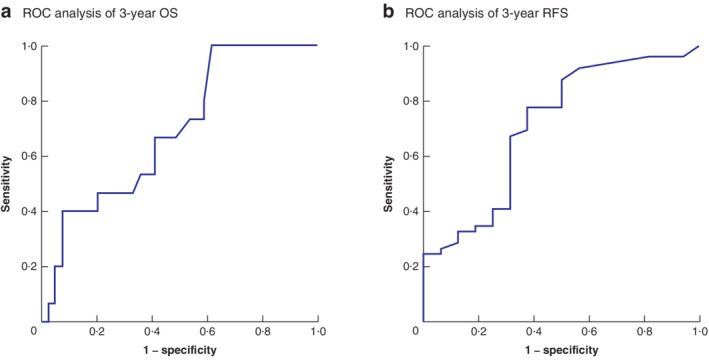
Receiver operating characteristic (ROC) curve analyses of total tumour volumes of resectable colorectal liver metastases before hepatectomy for prediction of survival after hepatic resection **a** Three‐year overall survival (OS); **b** 3‐year recurrence‐free survival (RFS). **a** Cut‐off value 100 ml; area under ROC curve (AUC) 0·691; sensitivity 40 per cent; specificity 93 per cent; *P* = 0·003. **b** Cut‐off value 10 ml; AUC 0·701; sensitivity 78 per cent; specificity 63 per cent; *P* = 0·085.

ROC curve analysis was performed to examine the association between TTV and both OS and RFS. Eleven patients whose observation period did not reach 3 years were excluded, so this analysis was done using the data of 83 patients. The analysis revealed that TTV was useful for predicting patient survival and tumour recurrence after initial hepatic resection (area under the ROC curve (AUC): 0·691 for OS and 0·701 for RFS) (*Fig*. 3).

Based on the ROC analysis, the TTV cut‐off value for OS was defined as 100 ml (sensitivity 40 per cent; specificity 93 per cent). A TTV of 100 ml is approximately 5·8 cm in diameter for a single tumour. The OS rate was significantly lower in patients with a TTV of at least 100 ml than in those with a TTV of less than 100 ml (*P* = 0·006). The 1‐, 3‐ and 5‐year OS rates of patients with a TTV of 100 ml or above were 73, 54 and 41 per cent respectively, compared with 97, 82 and 67 per cent in patients with a TTV below 100 ml.

The TTV cut‐off value for RFS was defined as 10 ml (sensitivity 78 per cent; specificity 63 per cent). A TTV of 10 ml is approximately 2·7 cm in diameter for a single tumour. The recurrence rate was significantly higher in patients with a TTV of at least 10 ml than in those with a TTV below 10 ml (*P* = 0·009). The 1‐, 3‐, and 5‐year RFS rates of patients with a TTV of 10 ml or more were 42, 17 and 14 per cent respectively, compared with 62, 58 and 58 per cent in patients with a TTV of less than 10 ml.

### Identification of predictive factors for recurrence‐free and overall survival

The associations of several variables with OS after hepatic resection for CRLM were investigated (*Table* 2). In univariable analysis, primary tumour location in the right colon, primary lymph node metastasis, bilobar liver metastasis and TTV of 100 ml or above were significantly associated with shorter OS. Multivariable analysis indicated that TTV of 100 ml or more was independently associated with poorer OS (hazard ratio (HR) 6·34, 95 per cent c.i. 2·08 to 17·90; *P* = 0·002); this factor also had the highest HR value for poor OS when compared with other significant factors, such as lymph node metastasis and bilobar lesions.

**Table 2:** Univariable and multivariable Cox proportional hazards analysis of factors associated with overall survival after initial hepatic resection in patients with resectable colorectal liver metastases

	Univariable analysis		Multivariable analysis	
	Hazard ratio	*P*	Hazard ratio	*P*
**Age > 64 years**	1·18 (0·55, 2·63)	0·668		
**Male sex**	0·99 (0·45, 2·39)	0·981		
**CEA level > 200 ng/ml**	1·95 (0·65, 1·54)	0·212		
**CA19‐9 level > 37 units/ml**	1·60 (0·74, 3·43)	0·223		
**MELD score**	0·99 (0·92, 1·07)	0·891		
**ICGR15 (>10%)**	0·71 (0·29, 1·56)	0·408		
**Primary tumour**				
Right colonic location	2·94 (1·28, 6·44)	0·012	3·55 (1·45, 8·44)	0·006
Depth of seroma invasion	1·59 (0·72, 3·39)	0·252		
Lymphatic invasion	0·78 (0·34, 1·81)	0·558		
Vessel invasion	0·54 (0·23, 1·34)	0·169		
Lymph node metastasis	6·41 (2·24, 27·00)	< 0·001	5·71 (1·70, 22·20)	< 0·001
**Metastases**				
Maximum diameter ≥ 5 cm*	2·05 (0·97, 4·27)	0·060		
Multiple tumour number	1·50 (0·71, 3·24)	0·288		
Bilobar tumour distribution	2·75 (1·29, 5·81)	0·010	5·61 (2·29, 14·30)	< 0·001
Metachronous timing of metastasis	0·97 (0·49, 2·06)	0·939		
Residual tumour	1·77 (0·73, 3·88)	0·187		
Preoperative chemotherapy	2·08 (0·97, 4·81)	0·060		
Partial response to preoperative chemotherapy (RECIST)	1·18 (0·48, 2·64)	0·698		
TTV ≥ 100 ml	3·02 (1·24, 6·67)	0·016	6·34 (2·08, 17·90)	0·002
Lobectomy	0·91 (0·48, 1·63)	0·746		

Values in parentheses are 95 per cent confidence intervals. *Total tumour volume (TTV) and maximum diameter were strong confounding factors, so only TTV was included in the multivariable analysis. When maximum diameter was used instead of TTV of 100 ml or more, the maximum diameter was also significantly associated with overall survival in the multivariable analysis (hazard ratio 2·91; 95 per cent c.i. 1·28 to 6·52; *P* = 0·011). CEA, carcinoembryonic antigen; CA, carbohydrate antigen; MELD, Model for End‐stage Liver Disease; ICGR15, indocyanine green retention rate at 15 min; RECIST, Response Evaluation Criteria In Solid Tumours.

With regard to RFS, primary tumour location in the right colon, primary lymph node metastasis and TTV of 10 ml or above were significantly associated with higher recurrence rates in univariable analysis (*Table* 3). In multivariable analysis, TTV of at least 10 ml was independently associated with poorer RFS (HR 1·90, 95 per cent c.i. 1·12 to 3·57; *P* = 0·017). The HRs for TTV of 10 ml or more, lymph node metastasis and primary right colonic lesion in relation to poor RFS were similar (*Table* 3).

**Table 3:** Univariable and multivariable Cox proportional hazards analysis of factors associated with recurrence‐free survival after initial hepatectomy in patients with resectable colorectal liver metastases

	Univariable analysis		Multivariable analysis	
	Hazard ratio	*P*	Hazard ratio	*P*
**Age > 64 years**	0·99 (0·59, 1·70)	0·948		
**Male sex**	1·03 (0·60, 1·86)	0·924		
**CEA level > 200 ng/ml**	1·58 (0·69, 3·15)	0·258		
**CA19‐9 level > 37 units/ml**	1·56 (0·92, 2·63)	0·094		
**MELD score**	0·98 (0·90, 1·05)	0·532		
**ICGR15 (>10%)**	0·72 (0·40, 1·27)	0·273		
**Primary tumour**				
Right colonic location	2·61 (1·43, 4·58)	0·002	2·60 (1·42, 4·61)	0·002
Depth of seroma invasion	1·67 (0·97, 2·81)	0·061		
Lymphatic invasion	1·01 (0·60, 1·71)	0·961		
Vessel invasion	0·98 (0·56, 1·80)	0·936		
Lymph node metastasis	1·94 (1·13, 3·48)	0·015	1·69 (0·95, 3·11)	0·025
**Metastases**				
Maximum diameter ≥ 5 cm	1·52 (0·87, 2·58)	0·138		
Multiple tumour number	1·51 (0·86, 2·55)	0·141		
Bilobar tumour distribution	1·25 (0·62, 2·33)	0·512		
Metachronous timing of metastasis	1·22 (0·70, 2·09)	0·470		
Residual tumour	1·21 (0·58, 2·31)	0·569		
Preoperative chemotherapy	0·84 (0·49, 1·41)	0·498		
Partial response to preoperative chemotherapy (RECIST)	0·90 (0·46, 1·66)	0·755		
TTV ≥ 10 ml	2·06 (1·19, 3·73)	0·009	1·90 (1·12, 3·57)	0·017
Lobectomy	0·78 (0·42, 1·38)	0·419		

Values in parentheses are 95 per cent confidence intervals. CEA, carcinoembryonic antigen; CA, carbohydrate antigen; MELD, Model for End‐stage Liver Disease; ICGR15, indocyanine green retention rate at 15 min; RECIST, Response Evaluation Criteria In Solid Tumours.

### Comparison of total tumour volume with other prognostic scores

In multivariable analysis, high TBS and Fong scores were not significantly associated with poorer OS (*P* = 0·095 and *P* = 0·948 respectively); only TTV was significantly associated with OS (*P* = 0·002) (*Table* 4).

**Table 4:** Multivariable Cox proportional hazards analysis of risk scores associated with overall survival

	Hazard ratio	*P*
Tumour Burden Score ≥ 3	2·72 (0·85, 12·10)	0·095
Fong score ≥ 3	0·98 (0·41, 2·27)	0·948
Total tumour volume ≥ 100 ml	6·34 (2·08, 17·90)	0·002

Values in parentheses are 95 per cent confidence intervals. Right colonic primary tumours, primary lymph node metastasis and bilobar liver metastasis were included in the model as confounding factors.

With regard to the association of other prognostic scores with RFS, high TBS and TTV were both significantly associated with poorer RFS (*P* = 0·037 and *P* = 0·017 respectively) (*Table* 5).

**Table 5:** Multivariable Cox proportional hazards analysis of risk scores associated with recurrence‐free survival

	Hazard ratio	*P*
Tumour Burden Score ≥ 3	2·01 (1·04, 4·19)	0·037
Fong score ≥ 3	1·71 (0·96, 3·02)	0·068
Total tumour volume ≥ 10 ml	1·90 (1·12, 3·57)	0·017

Values in parentheses are 95 per cent confidence intervals. Right colonic primary tumours and primary lymph node metastasis were included in the model as confounding factors.

### Survival and recurrence after hepatic resection based on total tumour volume

**Figure 4:**
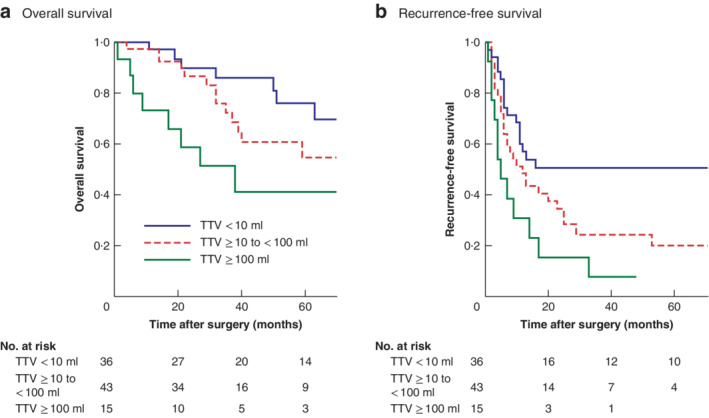
Kaplan–Meier analysis of overall and recurrence‐free survival after hepatic resection of resectable colorectal metastases according to total tumour volume **a** Overall and **b** recurrence‐free survival in three groups separated by total tumour volume (TTV) cut‐off values of 10 ml and 100 ml: TTV less than 10 ml (lowest volume), TTV 10 ml to less than 100 ml (mid volume), and TTV 100 ml or above (highest volume). **a** *P* = 0·283 (lowest *versus* mid volume), *P* = 0·016 (mid *versus* highest volume), *P* = 0·003 (lowest *versus* highest volume); **b** *P* = 0·038 (lowest *versus* mid volume), *P* = 0·085 (mid *versus* highest volume), *P* = 0·002 (lowest *versus* highest volume) (log rank test).

Patients were divided into three groups according to TTV cut‐off values for RFS and OS: less than 10 ml (36 patients), at least 10 ml to less than 100 ml (43), and 100 ml or more (15). The OS rate among patients with a TTV of at least 100 ml was significantly lower than that in patients with a TTV below 10 ml (*P* = 0·003) or with a TTV of 10 ml to less than 100 ml (*P* = 0·016) (*Fig*. 4 *a*). There was no significant difference in the OS rate between patients in the two lowest volume groups (*P* = 0·283).

In contrast, relative to patients in the lowest volume group, RFS rates were significantly lower among patients with a TTV of 100 ml or more (*P* = 0·002) and those with a TTV of 10 ml to less than 100 ml (*P* = 0·038) (*Fig*. 4 *b*). However, there was no significant difference in RFS rate between patients with a TTV of 100 ml or more and those with a TTV of 10 ml to less than 100 ml (*P* = 0·085).

**Figure 5:**
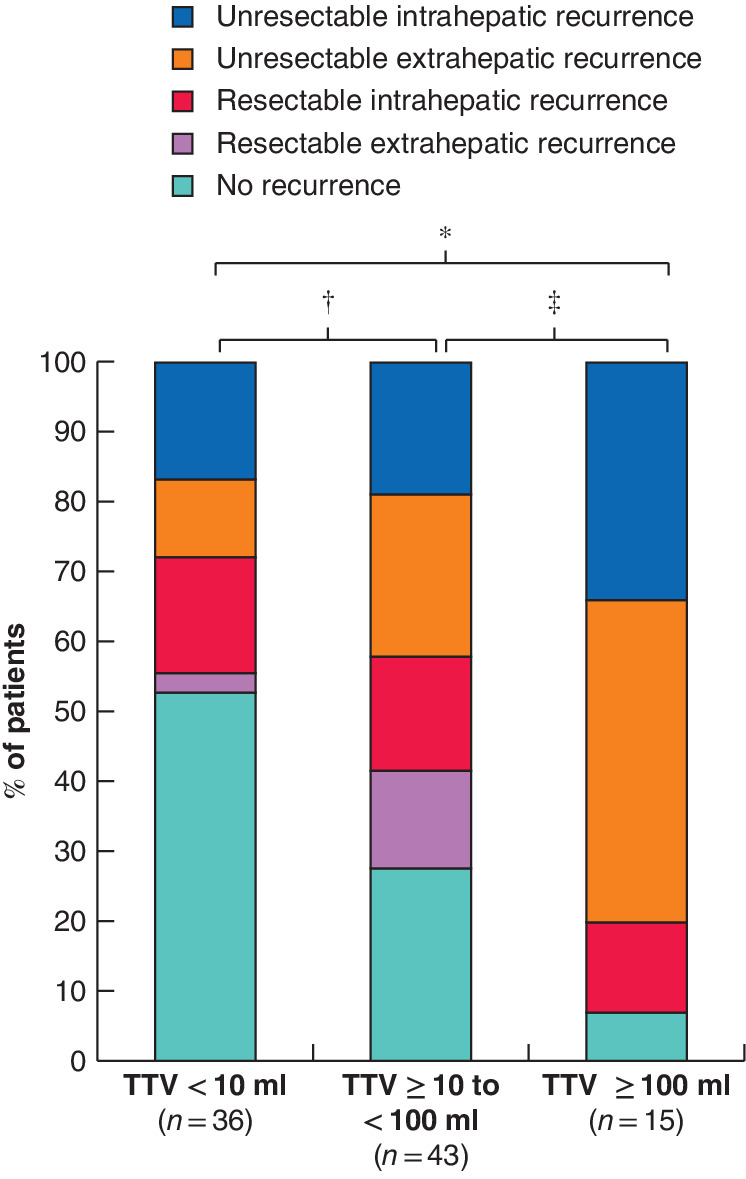
Bar chart of the proportions and sites of first recurrence after initial hepatic resection for colorectal liver metastases according to total tumour volume Some 28 per cent of patients (10 of 36) with a total tumour volume (TTV) lower than 10 ml had unresectable recurrences, 42 per cent of patients (18 of 43) with a TTV of 10 ml to less than 100 ml, and 80 per cent of patients (12 of 15) with a TTV of 100 ml or above. *P < 0·001, †*P* = 0·192 and ‡*P* = 0·009 (χ^2^).

In total, 17 of 36 patients with a TTV below 10 ml, 31 of 43 patients with a TTV of 10 ml to less than 100 ml, and 14 of 15 patients with a TTV of 100 ml or more had a recurrence after initial hepatic resection for CRLM. Ten of 36, 18 of 43, and 12 of 15 patients in these respective groups were considered unsuitable for surgery following an initial postoperative recurrence (those with intrahepatic multiple recurrences or extrahepatic recurrences except the lung) (*Fig*. 5). Patients with a TTV of 100 ml or above had a significantly higher rate of unresectable primary recurrences after initial hepatic resection than patients with a TTV of less than 10 ml (*P* < 0·001) and those with a TTV of 10 ml to less than 100 ml (*P* = 0·009).

## Discussion

Recent advances in diagnostic imaging have enabled direct measurement of TTV^32^, ^33^. In particular, three‐dimensional DCE‐CT images and simulation software may quantify precisely the irregular shapes of CRLM. These technologies were used in the present study, and revealed independent associations between the TTV and OS and RFS after hepatic resection in patients with CRLM. Moreover, a huge colorectal metastasis, defined using the TTV, was associated with unresectable recurrence after hepatic resection in these patients. The present results indicate that TTV is a good prognostic indicator of resectable CRLM, and suggest that it may be a suitable indicator of eligibility for hepatic resection in patients with CRLM.

In this study, TTV was the best predictor of OS after hepatic resection for CRLM, and had a higher HR than other well reported prognostic factors and scores, such as the primary lymph node metastasis status, primary tumour location and maximum tumour diameter, and TBS and Fong score. This finding may be biologically plausible, as TTV may directly reflect the tumour burden, and the total tumour cell number in particular. Previous studies^12^, ^14^, ^15^, ^16^, ^34^, ^35^ identified maximum tumour diameter and number of metastases as prognostic factors for hepatic resection in patients with CRLM. Maximum tumour diameter and number of metastases were used as alternative indices for TTV, as it is difficult to measure the TTV directly on CT images using conventional two‐dimensional software. However, assuming that the tumour is spherical, the volume is proportional to the cube of the diameter; therefore, maximum diameter does not accurately indicate the difference between the burden of large and small tumours (and may underestimate that of large tumours). In addition, as all malignant tumours are not spherical, an accurate assessment of tumour burden is more difficult. In the case of multiple metastases, evaluation of the maximum diameter assesses only one tumour, and so the total tumour burden may be underestimated. In this study, maximum tumour diameter was a prognostic factor for OS when used instead of TTV in multivariable analysis. However, the HR of maximum tumour diameter was inferior to that of TTV (2·91 *versus* 6·34 respectively). In addition, the TBS and Fong score, including maximum diameter and number, also had less prognostic value than TTV in the present study. The authors speculate that this could explain why TTV was the strongest prognostic factor for OS. Unlike in the past, recent advances in image‐processing technology mean that TTV can be measured easily using dedicated software.

A recent study^36^ of patients with chemorefractory metastatic colorectal cancer found that those with a high metabolically active tumour volume, as measured via fluorodeoxyglucose (FDG)‐PET–CT, had a significantly shorter OS. Although that study was similar to the present one, the analysis included extrahepatic metastases and detected only FDG‐PET‐positive lesions.

Among the three TTV groups, patients with a TTV below 10 ml had a significantly lower recurrence rate than patients with a TTV of 10 ml or above, and a significantly higher OS rate than those with a TTV of 100 ml or more. These results were logical, as, generally, a low recurrence rate leads to good survival. Notably, patients with a TTV of 10 ml to less than 100 ml and those with a TTV of 100 ml or more had similar recurrence rates, but the OS rate was significantly poorer in patients with a TTV of at least 100 ml. A possible explanation for this dichotomy may involve the pattern of recurrence. The proportion of patients with unresectable initial recurrences after hepatic resection was higher in the group with a TTV of 100 ml or above than that in the group with a TTV of 10 ml to less than 100 ml. In particular, 80 per cent (12 of 15) of patients in the former group developed unresectable recurrences after initial hepatic resection for CRLM, compared with only 42 per cent (18 of 43) in the latter group. This could be because patients with a TTV of 100 ml or more tend to develop secondary metastases from CRLM. Tumour cells from huge CRLM can easily invade the hepatic vasculature, but cannot be filtered by the liver, similar to colonic cancer cells, and are likely to cause multiple systemic metastases. The high rate of unresectable recurrence after hepatic resection and poor OS in patients with a TTV of 100 ml or above may trigger debate regarding the indications of hepatic resection for CRLM. In view of these observations, patients with such a high TTV are likely to be suitable for chemotherapy rather than upfront hepatic resection, even if the tumour is technically resectable.

Several limitations of this study should be acknowledged. First, it was a relatively small retrospective study of patients from a single institution. Second, significant heterogeneity was observed among the patient characteristics, such as differing perioperative chemotherapy regimens, timing of surgery, and indications. Third, TTV was measured on images acquired via DCE‐CT. Although multiphasic CT can detect most CRLM lesions, gadoxetic acid‐enhanced MRI may be preferable as it detects lesions more accurately^37^, ^38^. Lastly, the timing of hepatic resection was not standardized among the patients. Despite these limitations, this study has revealed an association between the preoperative TTV and prognosis in patients with resectable CRLM. These findings need further validation in large‐scale prospective studies, adjusted for the above‐mentioned limitations.

TTV may be a universal predictor of prognosis in patients with resectable CRLM. It was found to be the strongest predictor of survival and could predict an unresectable initial recurrence after first‐line hepatic resection. TTV may therefore be useful for identifying good candidates for hepatic resection.

## Disclosure

The authors declare no conflict of interest.
